# Caution: fundamental data quality issues underlying intelligent prediction of enzyme optimal pH

**DOI:** 10.1093/bib/bbag543

**Published:** 2026-09-29

**Authors:** Peng Zhou, Xuzhong Fang

**Affiliations:** State Key Laboratory of Submarine Geoscience, Second Institute of Oceanography, Ministry of Natural Resources, Baochu North Road, Xihu District, Hangzhou 310012, China; Key Laboratory of Marine Ecosystem Dynamics, Second Institute of Oceanography, Ministry of Natural Resources, Baochu North Road, Xihu District, Baochu North Road, Xihu District, Hangzhou 310012, China; School of Aeronautics and Astronautics, Shanghai Jiao Tong University, Dongchuan Road, Minhang District, Shanghai 200240, China

**Keywords:** enzyme optimal pH, sequence-based prediction, data quality, enzyme engineering, deep learning

To the Editor,

Deep learning-based prediction of enzyme optimal pH (pH_opt_) has become an efficient and cost-effective approach to advance enzyme engineering, complementing labor-intensive experimental assays. Qiu *et al*. [1] recently developed Seq2pHopt, a deep learning predictor of enzyme pH_opt_. While investigating the dataset used by Seq2pHopt, we have identified several data-related issues. The dataset employed in the development of Seq2pHopt was sourced from the recently proposed EpHod model, a profound framework for pH_opt_ prediction [1, 2]. EpHod directly predicts enzyme pH_opt_ from protein sequences utilizing embeddings from the ESM-1v protein language model (PLM), achieving a root mean squared error (RMSE) of 1.25 pH units on the held-out test set [2]. Via algorithm optimizations, Qiu *et al*. [1] subsequently used the EpHod pH_opt_ dataset to develop Seq2pHopt, which improved pH_opt_ prediction to an RMSE of 0.88 pH units. The sequence-based deep learning approaches enable rapid prediction of enzyme pH_opt_. Nevertheless, inherent annotation flaws in this dataset undermine the reliability of these predictive models.

The EpHod pH_opt_ dataset comprises 9855 enzymes annotated with pH_opt_ values from the BRENDA database [2]. This dataset showed severe label imbalance: nearly 75% of the pHopt values fall between pH 6 and 8, and 94% range from pH 5 to 9 [2]. To alleviate this label imbalance, measurements have been used to reweight the loss function with values inversely proportional to the label density, thus directing the models to focus on sparse extreme regions [2]. Given that accurate prediction for extremely acidic and alkaline enzymes is highly valuable for biological design and synthesis, the 28 entries with extreme pH_opt_ values (≥ 11.5 or ≤ 2.5) from the EpHod pH_opt_ dataset were cross-checked for experimental evidence from published literature. The pH_opt_ annotations of six entries were found notably inconsistent with experimentally determined results (Table 1), spanning the training, validation and test subsets. Detailed case analyses are presented below.

**Table 1:** Six erroneous pH_opt_ annotations in the EpHod dataset.

UniProt accession number	pH_opt_ in EpHod dataset	pH_opt_ from literature	Dataset subset
B5LXD7	11.5	1.0–1.5 [4]	Validation
A0A2Z5VGL1	2.4	experimental artefact [6]	Training
A0A2Z5VGM9	2.4	experimental artefact [6]	Training
A0A2Z5VGN0	2.4	experimental artefact [6]	Testing
Q6HA05	2	2.5 [7]	Training
Q97VS7	1.8	Not found	Testing

B5LXD7 is annotated with a pH_opt_ value of 11.5 [2, 3]. However, biochemical assays of the purified recombinant enzyme expressed in *Pichia pastoris* confirm its actual pH_opt_ ranges from 1.0 to 1.5 [4]. This striking discrepancy merits close attention. One possible explanation is that this misannotation occurred during annotation by BRENDA.

Three entries (A0A2Z5VGL1, A0A2Z5VGM9, and A0A2Z5VGN0) are uniformly annotated with a pH_opt_ value of 2.4 in the EpHod dataset. However, A0A2Z5VGL1, A0A2Z5VGM9, and A0A2Z5VGN0 were previously reported to exhibit specific ENGase activity toward fucose-containing oligosaccharides [5], yet follow-up experiments showed that these proteins lacked such activity [6].

Q6HA05 (Pepsin A1 in the Antarctic notothenioid *Trematomus bernacchii*) is assigned a pH_opt_ value of 2.0 in the EpHod training dataset, whereas biochemical characterization of the purified recombinant Pepsin A1 expressed in *Escherichia coli* yielded a pH_opt_ value of 2.5 [7]. Since *T. bernacchii* possesses two pepsins, A1 and A2, and Pepsin A2 has a pH_opt_ value of 2.0 [7], one possible explanation is that the pH_opt_ value of Pepsin A2 was annotated to Pepsin A1 by mistake.

Q97VS7 encodes endo-1,4-β-glucanase CelB (genomic locus: *SSO2534*), which is annotated with a pH_opt_ value of 1.8 in the EpHod test set. However, available literature only verifies the cellulase activity of Q97VS7 at pH 5.8 [8] with no experimental evidence supporting a pH_opt_ of 1.8. One possible explanation is that, within the BRENDA database, Q97VS7 was incorrectly annotated with the pH_opt_ value of another endo-β-glucanase (SSO1949). The genome of *Saccharolobus solfataricus* (strain ATCC 35092, formerly *Sulfolobus solfataricus*) harbors three putative endo-β-glucanases, located at genomic loci *SSO2534*, *SSO1949*, and *SSO1354*, among which SSO1949 has a well-validated pH_opt_ of 1.8 [9]. No conclusive data confirm that Q97VS7 has the same pH_opt_ value as SSO1949. Further experimental validation is required to verify the true pH_opt_ of Q97VS7.

After manually cross-checking 28 extreme-pH_opt_ entries in the EpHod dataset against literature and identifying six erroneous annotations, these data flaws raise critical scientific concerns regarding the performance and reliability of Seq2pHopt and EpHod when predicting pH_opt_ of extremely acidic and alkaline enzymes. Moreover, besides these errors in extreme-pH annotation, the vast majority of entries in the full dataset remain unverified, and additional unrecognized annotation errors likely exist. Importantly, in machine learning research, a rigorously curated and fully validated dataset serves as the indispensable cornerstone of robust and trustworthy predictive modeling, whereas low-quality data may bias model training and evaluation, undermine the reliability of prediction accuracy, and even trigger negative consequences [10]. On one hand, BRENDA undergoes continuous quality control to improve the accuracy of its content [11]. On the other hand, we suggest that data users should inspect datasets prior to usage and employ data that are as error-free as possible, even though such data-checking work is time-consuming and labor-intensive. The focus of this letter is neither on the measures taken to mitigate the impacts of annotation errors, nor on evaluating whether correcting these errors would substantially improve predictive performance. Instead, this letter calls for systematic inspection of the pH_opt_ dataset to support continuous improvements in enzyme-pH_opt_-prediction accuracy and facilitate the rational design and synthesis of high-performance enzymes.

**Key Points**

- Six clear errors were identified among 28 extreme-pH entries from the benchmark BRENDA-derived enzyme pH_opt_ dataset.
- These data flaws raise scientific concerns over the performance and reliability of enzyme pH_opt_ predictive models.
- This letter emphasizes systematic inspection of the benchmark dataset to improve the reliability of enzyme pH_opt_ prediction.

## Data Availability

No new data were generated in this study. The letter refers to the data provided by previous studies [1, 2]. Data was downloaded from Zenodo at https://doi.org/10.5281/zenodo.14252615.
